# Profiles of VGF Peptides in the Rat Brain and Their Modulations after Phencyclidine Treatment

**DOI:** 10.3389/fncel.2017.00158

**Published:** 2017-06-02

**Authors:** Barbara Noli, Fabrizio Sanna, Carla Brancia, Filomena D’Amato, Barbara Manconi, Federica Vincenzoni, Irene Messana, Maria R. Melis, Antonio Argiolas, Gian-Luca Ferri, Cristina Cocco

**Affiliations:** ^1^Neuro-Endocrine-Fluorecence (NEF) Laboratory, Department of Biomedical Sciences, University of CagliariMonserrato, Italy; ^2^Neuropsychobiology Laboratory, Department of Biomedical Sciences, University of CagliariMonserrato, Italy; ^3^Department of Life and Environmental Sciences, University of CagliariMonserrato, Italy; ^4^Institute of Biochemistry and Clinical Biochemistry, Catholic UniversityRome, Italy; ^5^Institute of Chemistry of the Molecular Recognition, National Research Council (CNR)Rome, Italy

**Keywords:** VGF peptides, schizophrenia, brain, nucleus accumbens, prefrontal cortex, phencyclidine, PPI response

## Abstract

From the VGF precursor protein originate several low molecular weight peptides, whose distribution in the brain and blood circulation is not entirely known. Among the VGF peptides, those containing the N-terminus portion were altered in the cerebro-spinal fluid (CSF) and hypothalamus of schizophrenia patients. “Hence, we aimed to better investigate the involvement of the VGF peptides in schizophrenia by studying their localization in the brain regions relevant for the disease, and revealing their possible modulations in response to certain neuronal alterations occurring in schizophrenia”. We produced antibodies against different VGF peptides encompassing the N-terminus, but also C-terminus-, TLQP-, GGGE- peptide sequences, and the so named NERP-3 and -4. These antibodies were used to carry out specific ELISA and immunolocalization studies while mass spectrometry (MS) analysis was also performed to recognize the intact brain VGF fragments. We used a schizophrenia rat model, in which alterations in the prepulse inhibition (PPI) of the acoustic startle response occurred after PCP treatment. In normal rats, all the VGF peptides studied were distributed in the brain areas examined including hypothalamus, prefrontal cortex, hippocampus, accumbens and amygdaloid nuclei and also in the plasma. By liquid chromatography-high resolution mass, we identified different intact VGF peptide fragments, including those encompassing the N-terminus and the NERPs. PCP treatment caused behavioral changes that closely mimic schizophrenia, estimated by us as a disruption of PPI of the acoustic startle response. The PCP treatment also induced selective changes in the VGF peptide levels within certain brain areas. Indeed, an increase in VGF C-terminus and TLQP peptides was revealed in the prefrontal cortex (*p* < 0.01) where they were localized within parvoalbumin and tyrosine hydroxylase (TH) containing neurons, respectively. Conversely, in the nucleus accumbens, PCP treatment produced a down-regulation in the levels of VGF C-terminus-, N-terminus- and GGGE- peptides (*p* < 0.01), expressed in GABAergic- (C-terminus/GGGE) and somatostatin- (N-terminus) neurons. These results confirm that VGF peptides are widely distributed in the brain and modulated in specific areas involved in schizophrenia.

## Introduction

Schizophrenia is a serious psychiatric illness with an occurrence of 1% in the world population (Rössler et al., 2005). One of the main hypotheses about the cause is the glutamatergic neuronal dysfunction (Carlsson et al., 1997). Indeed, the treatment of phencyclidine (PCP), which is a non-competitive N-methyl-D-aspartate (NMDA) receptor antagonist, simulates certain schizophrenia symptoms (Javitt and Zukin, 1991). Deficient prepulse inhibition (PPI) of the acoustic startle responses in schizophrenia patients is due to damage of sensorimotor pathways. In rats, PPI is damaged by treatments with different neurotransmitters such as dopamine agonists, serotonin agonists, or glutamate antagonists other than by an assortment of different manipulations involving neuronal pathways within the limbic cortex, ventral and dorsal striatum, pallidum, and pontine reticular areas (Mouri et al., 2007; Mena et al., 2016). The vgf (non acronymic) is a neutrophin-induced gene encoding for a primary product, VGF precursor or pro-VGF, composed of 617/615 amino acids (rat/mouse and human respectively) discovered because of a selective up-regulation by NGF in the rat pheochromocytoma cells (Levi et al., 1985). Chromosomal mapping has assigned human VGF to chromosome 7q22 (Canu et al., 1997) and mouse/rat Vgf to chromosome 5 (Salton et al., 1991). The VGF precursor protein is widely expressed in the brain and is processed to give rise to a number of peptides of low molecular weight. Often, the four N-terminal amino acids (in single letter code) and the peptide length are used to name the VGF peptides. Several VGF peptides have been identified as being related to metabolic mechanisms as is the case of the TLQP-21 peptide belonging to the TLQP peptide family (Bartolomucci et al., 2006; Brancia et al., 2010) and the so previously named NERP (neuroendocrine peptide) family that includes NERP-1, -2 (Yamaguchi et al., 2007; D’Amato et al., 2012; Melis et al., 2012), -3 (QQET-30; Fujihara et al., 2012) and -4 (NAPPE-19; D’Amato et al., 2015). Further functions related to neuronal circuits were also discovered for certain VGF peptides. Central administrations of a peptide called AQEE-30 into the hypothalamus, produces penile erection probably by activating nitric oxide (NO) and oxytocin (Succu et al., 2004, 2005). Indeed, another peptide belonging to the TLQP family and called TLQP-62 is involved in hippocampal neuronal circuits related to psychiatric diseases (Thakker-Varia et al., 2010), and depression (Thakker-Varia et al., 2007; Lin et al., 2015). Both TLQP-21 and TLQP-62 seem to be involved in neuronal regulation of the rat oestrus cycle (Noli et al., 2014) and hamster photoperiodic mechanisms related to cholinergic neurons of the brain cortex (Noli et al., 2015). In the Alzheimer’s disease patients, the VGF peptides named “GGGE” were found decreased in the cerebro-spinal fluid (CSF; Carrette et al., 2003) while those named PGH, together with the VGF C-/N- terminus peptides and the NERP-1 were reduced in the brain cortex (Cocco et al., 2010). The VGF C-/N- terminus peptides have also been related to the amyotrophic lateral sclerosis mechanisms (Zhao et al., 2008; Brancia et al., 2016). As to schizophrenia patients, one VGF N-terminal fragment (VGF_24–60_) was found to be increased in the CSF from drug naïve and treated subjects (Huang et al., 2006, 2007), while in post mortem hypothalamus samples, immunoreactivity cell density for the VGF N-terminus was reduced in neurons of the paraventricular and supraoptic nuclei (Busse et al., 2012). Several loci have been related to schizophrenia, including the 7q22, but unfortunately, the analysis of the regional candidate VGF gene, revealed no significant association to the clinical diagnosis of schizophrenia (Wedenoja et al., 2010). Although some VGF peptides have crucial roles in neurodegenerative and neuronal conditions, their localization in the brain is not completely known. In particular, the available information regarding VGF peptides and schizophrenia is limited to the human hypothalamus/CSF and restricted to the VGF N-terminal fragment with pending information coming from animal models and other VGF fragments. Therefore, we aimed to better investigate the involvement of the VGF peptides in schizophrenia by studying their localization in specific brain regions, crucial for the disease, and revealing their possible modulations in response to certain neuron alterations occurring in schizophrenia. We used a schizophrenia rat model in which alterations in the PPI of the acoustic startle response occurred after PCP treatment (Mouri et al., 2007). Indeed PCP is a glutamate receptor antagonist of the NMDA subtype, which causes NMDA receptor hypoactivity (Meador-Woodruff et al., 2003) and mimics both the positive and negative symptoms of schizophrenia in humans (Slavney et al., 1977; Allen and Young, 1978; Javitt and Zukin, 1991; Steinpreis, 1996). Antibodies against sequences encompassing the C-/N- terminus, TLQP-, and GGGE- peptides, as well as the so named NERP-3 and -4, were produced and used either to carry out specific immunosorbent assays (ELISA) or for immunolocalization studies. High-pressure performance liquid chromatography-mass spectrometry (HPLC-MS) was also used to confirm their amino acid sequences. The VGF fragments to be studied were chosen for their previous involvement in schizophrenia (N-terminus), other neuronal conditions (GGGE and C-terminus) or hypothalamic mechanisms (NERPs and TLQP) that are also altered in many psychiatric diseases (Oliver et al., 2012; Tognin et al., 2012; Tiwari et al., 2013).

## Materials and Methods

### Animals

Male Sprague Dawley rats (250–300 g, Envigo Correzzana, Italy) were assembled four per cage (38 cm × 60 cm × 20 cm). Rats (*n* = 88), were taken under precise conditions (24°C, 60% humidity, reversed 12 h light/dark cycle, with lights off from 08:00 to 20:00 h), with water and standard food *ad libitum*, and then a group (*n* = 80) were used for the experiments, performed between 10:00–14:00 h accordingly to the guidelines of the European Communities Directive of September 22, 2010 (2010/63/EU) and the Italian Legislation (D.L. March 4, 2014, n. 26). The protocol has been approved by the Italian Ministry of Health (Authorization n.1237/2015-PR to MRM), and by the Ethical Committee for Animal Experimentation of the University of Cagliari, Italy.

### PCP Treatment and PPI Response

In order to validate the assumption that PCP treatment was effective in inducing behavioral changes resembling schizophrenia-like symptoms in our experimental conditions, i.e., it was effective in modifying PPI, of the acoustic startle response, 80 male rats were randomly assigned to five experimental conditions (*n* = 16/condition), each one with a different treatment regimen. Within each condition, half of the rats (*n* = 8) received PCP (purchased from R&D Systems Europe Ltd., Abingdon, UK 2.5 mg/kg, SC) or the corresponding volume of saline (1 mL/kg of rat body weight, SC; *n* = 8). The main conditions were as follows: acute treatment (animals were treated once); sub-chronic treatment (animals were treated once a day for eight consecutive days); chronic treatment (animals were treated once a day for 21 consecutive days). The acute treatment comprised animals that underwent the behavioral test (PPI assessment) after 10–15 min from the PCP injection. The sub-chronic and chronic treatment conditions comprised animals, which underwent the behavioral test (PPI assessment) 30 min after the last PCP injection (no wash out condition) and animals, which underwent the behavioral test 24 h after the last PCP injection (wash out condition). Treatments were carried out between 10:00 h and 13:00 h counterbalancing the sequence of PCP- and saline- treated rats. The day of the test, the rats in their home-cages were transferred during the dark phase of the cycle in a testing room under controlled environmental conditions (a sound proofed room lit by a dim red light). Each testing session was conducted with the same number of PCP treated rats and their matched saline treated controls. PPI testing was performed with minor modifications as already described (Frau et al., 2016). The apparatus used for detection of startle reflexes (Med Associates, St Albans, VT, USA) consisted of four standard cages placed in sound-attenuated chambers with fan ventilation. Each cage consisted of a plexiglas cylinder of 9 cm diameter, mounted on a piezoelectric accelerometric platform connected to an analog-digital converter. Two separate speakers conveyed background noise and acoustic bursts. Both speakers and startle cages were connected to a main computer, which detected and analyzed all chamber variables with specific software. Before each testing session, acoustic stimuli and mechanical responses were calibrated. The test began after acclimatization phase (5 min), with a 70 dB background white noise, continuing for the entire session. This period was followed by three blocks, comprising five pulse-alone trials of 115 dB (the first and the third), while the second includes a pseudorandom sequence of 50 trials, containing 12 pulse-alone trials, 30 trials of pulse preceded by 74, 78, or 82 dB pre-pulses (10 for each pre-pulse loudness level), and eight no-pulse trials, where exclusively the background noise was delivered. Time between two consecutive trials (inter-trial intervals) was arbitrarily selected between 10 s and 15 s. The % PPI was measured through the formula: % PPI = (100—mean startle amplitude for prepulse trials/mean startle for pulse alone trial) × 100.

### Statistical Analysis of Behavioral Data

Behavioral data from PPI experiments were analyzed by two way ANOVA. Although a trend toward a higher inhibition was observed with increasing tone intensities, we detected no significant differences in PPI responses to the three different prepulse intensities, therefore data from the three different prepulse intensities were put together for each rat to calculate individual mean values which were used for statistical analyses conducted by a two way ANOVA with the duration and the treatment as between subject factors. *Post hoc* pair wise contrasts on statistically significant main effects and/or interactions revealed by ANOVA analysis were obtained through Bonferroni test. Statistical analyses were applied using PRISM, Graph Pad 5 Software (San Diego, SA, USA) and the significance level were always *p* < 0.05.

### Tissue Collection

Brain tissues from rats that did not receive any treatment nor were exposed to the PPI procedure were also used both for immunohistochemistry (*n* = 4) as well as for the determination of amino acid sequence by HPLC-MS (*n* = 4). Irrespective of the fact that rats underwent or not the PPI procedure and/or the PCP treatment, for ELISA at sacrifice, rats underwent deep diethyl ether anesthesia, blood (approximately 2 ml) was drawn by cardiac puncture, hence rats were rapidly decapitated for tissue extraction. The brain slide samples (coronal sections of 2 mm) were obtained using a cooled rat brain matrix through razor blades. The samples from different brain areas (hypothalamus, ventral and dorsal hippocampus, amigdaloid and accumbens nuclei, prefrontal cortex) were dissected from the brain slides using punches of 3, 4 and 5 mm dimensions as appropriate, following the coordinates of the Paxinos Atlas of the rat brain (Paxinos and Watson, 2007). Tissue samples were extracted (~10 ml/g wet tissue) in ice-cold phosphate buffer saline (PBS: 0.01 mol/L, pH 7.4) containing protease inhibitor cocktail (PIC, 5 m/ml in PO4 buffer, 0.01–0.05 M, pH 7.2–7.4 + NaCl 0.15 MP8340, Sigma-Aldrich, Schnelldorf, Germany), homogenized for 3 min using a micro sample pestle, hence tubes were heated for 10 min, and centrifuged (3000 rpm, 15 min). Supernatants were stored frozen until use (−30°C). Blood samples were collected in a tube containing ethylenediaminetetraacetic (EDTA: 1.78 mg/ml), rapidly centrifuged (11,000 rpm, 5 min), and plasma was stored frozen (−30°C) until analysis. For HPLC-ESI-MS, brain samples pooled from four untreated male rats were extracted in PBS containing PIC, homogenized by an Ultra-Turrax Homogenizer (Ika-Werke, Staufen, Germany), heated in a boiling water bath (10–15 min), and centrifuged (3000 rpm, 10–15 min). The supernatant was transferred on centrifugal filters with the molecular weight cut-off of 10 kDa and 3 kDa (Amicon Ultra device, Merck Millipore, Tullagreen Carrigtwohill Co. Cork, Ireland) and the resulting filtrate was lyophilized and stored frozen. Differently from ELISA and HPLC-MS, for immunohistochemistry, rats were anesthetized using chloral hydrate (400 mg/kg i.p.) and perfused-fixed with 4% paraformaldehyde in 0.1 M phosphate buffer, pH 7.2–7.4.

### VGF Antisera

The VGF N- and C- terminus, TLQP, NERP-3 and NERP-4 (also named NAPPE) antisera were produced as previously reported (Ferri et al., 1995; Brancia et al., 2005; Cocco et al., 2010; D’Amato et al., 2015), while in order to obtain the VGF_375–407_ antiserum, the N-terminal decapeptide (GGGEDEVGEE), was conjugated with bovine thyroglobulin, via an additional C-terminal cysteine and used for immunizations. Specificity of each antiserum was achieved through ELISA (Table 1). It is relevant to point out here that our VGF antibodies, raised against truncated sequences at their N- or C- terminus, are expected to cross-react also with extended peptides. We assume that TLQP-62 will react in the TLQP assay much as TLQP-21 and in a way comparable to the VGF C-terminus standard in the relevant assay. Such assumption appears to fit well with our previous studies of TLQP-peptides, based on gel chromatography coupled with ELISA (Brancia et al., 2010, 2016; Noli et al., 2014, 2015). In some tissues, a large MW form bona fide corresponding to VGF (found in the void volume upon sephadex chromatography) was recognized by both the VGF N-terminus and C-terminus assays (Brancia et al., 2010; Cocco et al., 2010; Noli et al., 2014).

**Table 1:** VGF assay characterization.

Antisera	Peptide	IC_50_ pmol/ml	CV1 %	CV2 %	Cross-reactivity %
**rVGF C-term.**	rVGF_609–617_ (IEHVLLHRP)^1^	0.02	2–3	8–10	100
	hVGF_607–615_				<0.01
	hVGF_603–612_				<0.01
**TLQP**	rVGF_556–564_ (TLQPPASSR)^2^	1.1	3–5	10–13	100
	rVGF_555–564_ R-TLQPPASSR*				3.5
	rVGF_556–566_ (TLQP-11)				122
	rVGF_556–576_ (TLQP-21)				183
**rVGF N-term.**	rVGF_24–31_ (APPGRSDVYP)^ 3^	10	3–7	9–12	100
	hVGF_23–30_				<0.001
	mVGF_24–31_				<0.001
**NERP-4**	rVGF_489–497_ (NAPPEPVPP)^ 4^	6	5	6–9	100
	rVGF_489–507_				95
	rVGF_488–496_				<0.001
**NERP-3**	rVGF_197–206_ (LESPGPERVW)^ 5^	10	8	5–8	100
	rVGF_197–207_				<0.001
	hVGF_177–206_				90
	hVGF_199–206_				98
**GGGE**	rVGF_375–382_ (GGGEDEVG)^ 6^	0.6	3	8–10	100
	hVGF_373–380_				<0.001
	rVGF_375–420_				150

IC_50_: concentration of peptide producing 50% inhibition of the maximum signal; CV1 and CV2: intra- and inter-assay variation, respectively; h: human; r: rat. ^1–6^“reference” peptides, used for immunization, plate coating, and as measurement standards, respectively. *Arg-extended: an Arginine residue was added at the peptide N-terminus, to mimic its extended sequence within the VGF precursor. The cross-reactivity of each peptide was expressed compared to the “reference” peptide used in the assay, hence a >100% reactivity is indicated for peptides with higher reactivity (e.g., TLQP-21).

### ELISA

Competitive ELISA was performed as previously reported (D’Amato et al., 2008; Cocco et al., 2010). For each assay characterization (Table 1), a standard curve was obtained using a range of concentrations of each of the peptides listed. The peptide corresponding to the immunogen was treated as “reference”, with a certain amount of such peptide producing a 50% inhibition of the maximum signal (50% inhibitory concentration), and showing 100% reactivity in the assay. The other peptides listed resulted in a standard curve parallel to the “reference” one, but variably shifted to the right (more peptide required to produce 50% inhibition, hence lower reactivity in the assay, i.e., <100% reactivity), or to the left (less peptide produced 50% inhibition, hence higher reactivity, >100% vs. reference peptide). Synthetic peptides were used for plate coating (Nunc, Milan, Italy) for 3 h at room temperature, then the wells were treated with PBS (containing 9% normal serum from the secondary antibody donor species, 20 nM aprotinin, and 1 mg/mL EDTA) for 2 h. Primary incubations, with the VGF antibodies, (dilution: 1:5000–1:12000) were carried out in duplicate, including serial standard dilutions in parallel with rat samples (brain or plasma; 3 h at room temperature). Biotinylated secondary antibodies (1:10000; Jackson, West Grove, PA, USA), streptavidin-peroxidase conjugate (Biospa, Milan, Italy), and tetramethylbenzidine (X-tra Kem-En-Tec, Taastrup, Denmank) as substrate were used to reveal the positive labeling. The reaction was stopped with hydrogen cloride (1 mol/L) and optical density was measured at 450 nm using a multilabel plate reader (Chameleon: Hidex, Turku, Finland). Recovery of synthetic peptide/s added to plasma, or to tissue samples at extraction was >85% for all assays used.

### Statistical Analysis of ELISA Data

Analysis of variance of ELISA data was carried out by one-way ANOVA, followed by *post hoc* multiple comparison tests (Student-Newman-Keul test), or by two-tailed Student’s *t*-test as appropriate by means of the StatistiXL software.

### HPLC High-Resolution ESI-MS and MS/MS Analysis

Freeze-dried material (5 μg) were re-suspended in 50 μl of aqueous formic acid (0.1% v/v) and 10 μl of the solution analyzed by an Ultimate 3000 RSLCnano system coupled with an Orbitrap Elite mass spectrometer (ThermoFisher, San Jose, CA, USA), using an EASY-Spray Column PepMap®RSLC C18 (3 μm particle diameter; 75 μm ID × 15 cm). Eluent solutions were constituted by: 0.1% (v/v) aqueous formic acid and acetonitrile with 0.1% (v/v) aqueous formic acid. The used gradient was linear from 0% to 55% of B in 35 min, at a flow rate of 300 nL/min. The Elite-Orbitrap mass spectrometer operated in data-dependent mode. Each full MS scan (60,000 resolving power) was followed by five MS/MS scans applied on the first five multiple-charged ions dynamically selected and disintegrated through collision-induced dissociation at a normalized collision energy of 35%. Thermo Proteome Discoverer 1.4 software was utilized to analyze Tandem mass spectra, while the SEQUEST cluster (University of Washington, Seattle, WA, USA, licensed by Thermo Electron Corp) was useful to search engine against UniProtKB Rattus norvegicus proteome (release 2016-03). The limits used for peptide matching were Xcorr scores greater than 1.5 for singly charged peptide ions while 2.0 for doubly charged ions and 2.5 for triply charged ions. Precursor mass search tolerance was set to 10 ppm, and fragment mass tolerance to 0.02 Da. The N-terminal modification (Gln → pyro-Glu) and the C-terminal amidation were selected as dynamic modifications. A false discovery rate (FDR) below 1% was applied. Peptide sequences and sites of covalent modifications were confirmed by manual spectra annotation by matching experimental MS/MS spectra with the theoretical ones generated by the MS-Product program available on Protein Prospector website^1^. The match was assumed as positive when: (1) all the experimental *m/z* values were higher than 5% in the theoretical fragmentation spectrum; and (2) experimental and theoretical values differed less than ±0.03 m/z.

### Immunohistochemistry

After fixation time, brains were included in an embedding medium (Cocco et al., 2003) and cut (at 10 μm) using a HM-560 cryomicrotome (Microm; Walldorf, Germany; Ferri et al., 2002). Sections comprehensive of the prefrontal cortex and the nucleus accumbens were incubated overnight in a humid chamber mixing the anti VGF antibodies (dilutions: 1:600–1:4000) with different neurotransmitters/enzymes/neuromodulators (Table 2), all diluted in PBS containing 30 ml/L of normal donkey serum, 30 ml/l of normal rat serum and 0.02 g/L NaN3. Relevant species-specific donkey secondary antibodies conjugated with Cy_3_ or Cy_2_ (Jackson Immunoresearch Laboratories, West Grove, PA, USA) were used (1:400). Slides were covered with PBS-glycerol (40%), observed and photographed using BX41 and BX51 fluorescence microscopes (Olympus, Milan, Italy) equipped with the Fuji S2 and S3 Pro digital cameras (Fujifilm, Milan, Italy). Routine controls included substitution of each antibody, in turn, with PBS, the use of pre-immune or non-immune sera, and the testing of each secondary antibody with their respective non-relevant primary antibodies.

**Table 2:** Antibodies used in immunohistochemistry.

Antigen	Diluition	Species	Producer/Reference
Calretinin	1:5000	Mouse	Millipore
r.GAD	1:1500	Mouse	DSHM
r.NOS	1:600	Sheep	Chemicon
h.NOS	1:800	Rabbit	Biomol international
NPY	1:700	Rabbit	Ferri et al. (1988)
r/h Parvoalbumin	1:10000	Mouse	Sigma
Somatostatin	1:800	Rabbit	Abcam
r/h TH	1:1000	Mouse	Sigma
r. TH	1:1000	Chicken	Millipore
r. VAChT	1:2000	Goat	ENZO

*GAD, glutamic acid decarboxylase; NOS, nitric oxide synthase; NPY, neuropeptide Y; TH, tyrosine hydroxylase; VAChT, vesicular acetylcholine transporter; r, rat; h, human*.

## Results

### VGF Peptide Levels in Normal Rats

**Figure 1:**
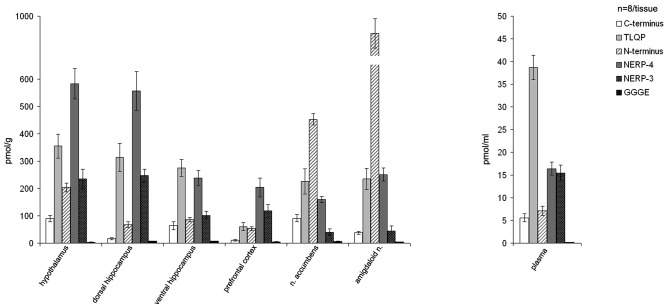
Content of the VGF peptides in the brain and plasma of male rats. VGF peptides were measured, by ELISA, in extracts from selected brain areas (hypothalamus, dorsal and ventral hippocampus, prefrontal cortex, nucleus accumbens and amygdaloid nucleus). Mean values (SEM) are from eight rats/tissue. Pmol/g: picomoles/grams.

In the normal rats, the VGF peptides were expressed in all the brain areas examined (hypothalamus, prefrontal cortex, nucleus accumbens, dorsal and ventral hippocampus, amygdaloid nucleus) and also in the plasma (Figure 1). VGF peptide levels ranged between approximately 4 pmol/g and 900 pmol/g in the brain and between 6 pmol/ml and 40 pmol/ml in plasma. Among the brain areas studied, the richest in the VGF peptides was the amygdaloid nucleus containing about 900 pmol/g, revealed with the N-terminus antibody. Analysis of each brain area for the content of the VGF peptides, revealed the following: the hypothalamus, dorsal hippocampus and prefrontal cortex contained more NERP-4, the accumbens and amygdaloid nuclei were more reactive to the N-terminus antibody, while the ventral hippocampus contained more TLQP peptides compared to the other VGF peptides. Conversely, in plasma, the TLQP peptides were the most represented.

### HPLC-MS Analysis of VGF Peptides

The RP-HPLC high-resolution ESI-MS analysis allowed the detection of 13 mass values attributed to different fragments of proVGF. Table 3 reports the experimental monoisotopic *m/z* values of the mono-charged peptides of proVGF, the post-translational modifications and the sequences determined by MS/MS experiments. Fragments VGF_24–54_ and VGF_24–60_ derived from the proVGF N-terminus, and fragments VGF_587–600_, VGF_588–600,_ derived from the C-terminus portion. Moreover, the following peptides belonging to the NERP family were identified: NERP-3 (VGF_180–209_), with the N-terminal Gln converted to Pyro-Glu, NERP1 (VGF_285–309_), amidated at the C-terminus and two fragments encompassing NERP-4 (VGF_489–507_ and VGF_487–507_). We unfortunately failed to identify the TLQP and GGGE peptides, however, we detected and characterized the same VGF_353–372_ fragment previously identified in the striatum (Karlsson et al., 2013), and its shorter form (VGF_360–372_).

**Table 3:** *m/z* values ([M+H]^+^, monoisotopic) of VGF peptides detected in rat brain by RP-high-pressure performance liquid chromatography (HPLC) high-resolution ESI mass spectrometry (MS) and confirmed by MS/MS experiments.

Position	Experimental [M+H]^+^	Modifications	Sequences
24–54	3242.58 (± 0.03)	–	APPGRSDVYPPPLGSEHNGQVAEDAVSRPKD
24–60	3868.88 (± 0.04)	–	APPGRSDVYPPPLGSEHNGQVAEDAVSRPKDDSVPEV
180–209	3390.67 (± 0.03)	N-Term (Q→pyro D)	QQETAAAETETRTHTLTRVNLESPGPERVW
238–282	4836.47 (± 0.05)	–	MSENVPLPETHQFGEGVSSPKTHLGETLTPLSKAYQSLSAPFPKV
285–309	2558.31 (± 0.03)	C-Term (Amidated)	LEGSFLGGSEAGERLLQQGLAQVEA
301–329	3150.61 (± 0.04)	C-Term (Amidated)	QQGLAQVEAGRRQAEATRQAAAQEERLAD
353–372	2461.08 (± 0.03)	–	GLQETQQERENEREEEAEQE
360–372	1391.55 (± 0.03)	–	ENEREEEAEQE
487–507	2171.21 (± 0.03)	–	KKNAPPEPVPPPRAAPAPTHV
489–507	1915.02 (± 0.03)	–	NAPPEPVPPPRAAPAPTHV
587–600	1700.83 (± 0.03)	–	RAQEEADAEERRLQ
588–600	1544.73 (± 0.03)	–	AQEEADAEERRLQ

*Numbering refers to the sequence of the protein with the signal peptide*.

### Effect of Acute, Sub-Chronic or Chronic PCP Treatment on the PPI Response

**Figure 2:**
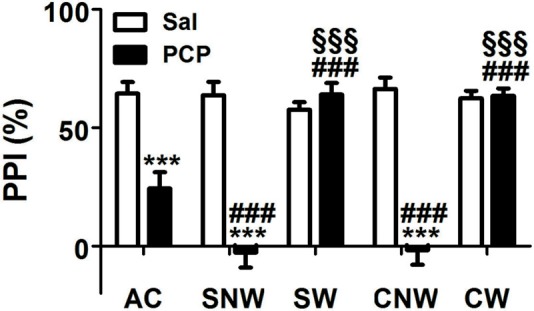
Effect of acute, subchronic or chronic PCP (phencyclidine) treatment on prepulse inhibition (PPI) response. Rats were treated once a day as well as once a day for 8 or 21 days with PCP (2.5 mg/kg, SC) or saline (1 mL/kg, SC) and tested for the PPI of the acoustic startle response 30 min or 24 h after the last drug treatment. AC, acute treatment; SNW and SW, subchronic treatment without or with 24 h wash out, respectively; CNW and CW, chronic treatment without or with 24 h wash out, respectively. Mean values (SEM) from 8 rats/group. ***p < 0.001 vs. the corresponding saline treated rats; ^###^*p* < 0.001 vs. acutely (1 day) treated rats; ^§§§^*p* < 0.001 vs. the corresponding no washed out rats (two way ANOVA followed by Bonferroni’s *post hoc* comparisons).

As shown in Figure 2, PCP treatment induced an impairment of PPI response after acute, sub-chronic and chronic treatment, with some differences in the entity of the effects due to the duration of the treatment regimen. Accordingly, two ways ANOVA revealed a significant effect of the drug (*F*_(1,110)_ = 107.2, *p* < 0.001), of the treatment regimen (F_(4,110)_ = 17.92, *p* < 0.001), and of interaction between the two factors (F_(4,110)_ = 24.46, *p* < 0.001). *Post hoc* comparisons revealed that acute, sub-chronic and chronic PCP treatments without wash out, all induced PPI impairment (24.33%, −2.62% and −1.60% for acute, sub-chronic and chronic treatment, respectively, compared to values between 57.68% and 66.49% of saline treated rats, all *p* < 0.001); in contrast, PCP-treated animals which underwent the 24 h wash out displayed PPI values indistinguishable from those of their saline matched controls (64.07% and 63.51% for sub-chronically and chronically PCP treated rats, respectively; all *p* > 0.05 when comparing PCP-washed out animals to the corresponding saline-treated rats, and all *p* < 0.001 when comparing PCP-washed out animals with the corresponding PCP not washed out rats). Finally, *post hoc* comparisons revealed also a significant difference in the entity of PPI impairment between acutely (24.33%) and both no wash out sub-chronically (−2.62%) and chronically (−1.60%) PCP-treated rats (both *p* < 0.001), with no difference in the entity of PPI impairment between no washed out sub-chronically and chronically treated rats (*p* > 0.05).

### VGF Peptide Levels in Saline- and PCP- Treated Rats

**Figure 3:**
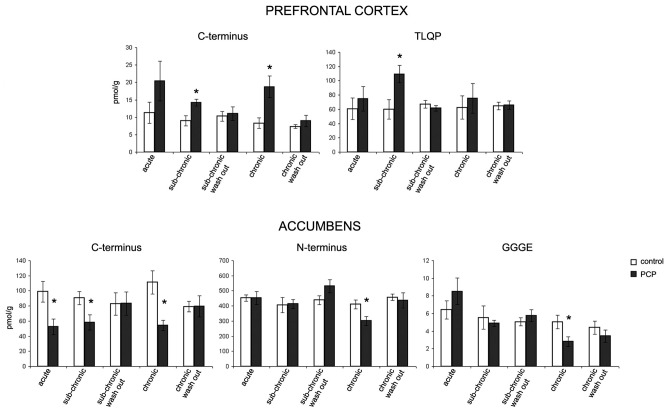
Effect of PCP treatment on the content of VGF peptides in the prefrontal cortex and nucleus accumbens. In the prefrontal cortex, we found an increase in VGF C-terminus levels after sub-chronic and chronic PCP treatment, as well as in TLQP peptides exclusively after sub-chronic PCP treatment when compared to the respective saline treated rats (*p* < 0.01). Conversely, in the nucleus accumbens, PCP treatment produced a decrease in the VGF C-terminus levels after all types of treatments (acute, sub-chronic and chronic), while N-terminus- and CGGE-peptide levels were reduced after chronic PCP treatment when compared to the respective groups of saline-treated rats (*p* < 0.01). The levels of all VGF peptides changed in PCP- (sub-chronic and chronic) treated rats returned to similar levels to those of saline-treated controls after 1 day from the last PCP treatment (sub-chronic and chronic wash out groups). Mean values are (SEM) from eight rats/group. *p < 0.01 vs. the corresponding saline-treated rats (one way ANOVA followed by Bonferroni’s *post hoc* comparisons). Pmol/g: picomoles/grams.

VGF peptide levels were measured in the prefrontal cortex, nucleus accumbens, dorsal and ventral hippocampus, amygdaloid nucleus and hypothalamus as well as in plasma of saline- and PCP- treated rats that underwent the PPI test under the acute, sub-acute and chronic treatment condition, with the aim of unraveling possible changes in the levels of one or more VGF peptides (Figure 3). We found changes in the content of VGF peptides exclusively within the prefrontal cortex and nucleus accumbens but not in the other brain areas studied or in plasma (not shown). In the prefrontal cortex, we found an increase in VGF C-terminus after sub-chronic and chronic PCP-treated rats, as well as for TLQP peptides exclusively in the sub-chronic group when compared to the respective saline treated rats (*p* < 0.01). Conversely, in the nucleus accumbens, PCP treatment produced a decrease in VGF C-terminus levels after all types of treatment (acute, sub-chronic and chronic), while N-terminus- and GGGE-peptides were reduced after chronic PCP treatment when compared to the respective groups of saline-treated rats (*p* < 0.01). In both the prefrontal cortex and nucleus accumbens, the amount of all peptides changed after PCP-treatment, returned to similar levels to those of saline-treated controls after 1 day from the last PCP treatment (sub-chronic and chronic wash out groups).

### VGF Peptide Localization in Brain

**Figure 4:**
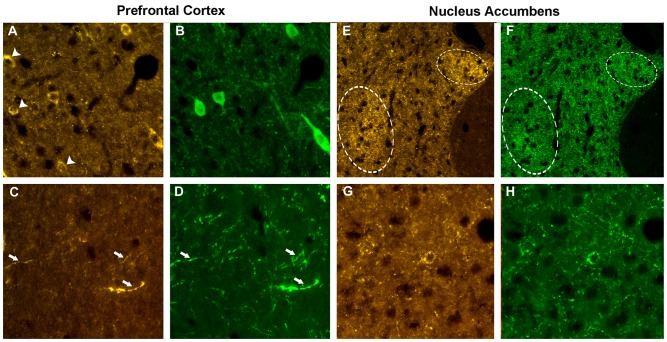
Phenotype of the VGF positive neurons. Prefrontal cortex. Using the VGF C-terminus **(A)** and the parvoalbumin **(B)** antibodies, an incomplete colocalization profile was found in the cell bodies, with some perikarya labeled by the VGF C-terminus antibody only (identified by the arrows, **A**). Almost all the TLQP positive nerve axons **(C)** contained also tyrosine hydroxylase (TH; **D**, identified by the arrows). Nucleus accumbens. C-terminus-immunoreactivity **(E)** was revealed in a subpopulation of neuron terminals containing glutamic acid decarboxylase (GAD; **F**) inside specific areas of the shell (identified by the circles). The N-terminus immunoreactivity **(G)** was almost completely found in all axons-, perikarya- and nerve terminals-secreting somatostatin **(H)**. VGF antibody: Cy3 red labeling, parvoalbumin, somatostatin, GAD: Cy2 green labeling. Magnification: 600× **(A–D,G,H)** and 400× **(E,F).**

The localizations of the VGF peptides, whose levels were changed by ELISA in the prefrontal cortex and nucleus accumbens after PCP treatment, were studied in the same areas of normal rats, to characterize the neuronal circuits containing the VGF peptides. We used VGF antibodies together with antibodies against the relevant neurotransmitters, enzymes or neuromodulators (Table 2). In the prefrontal cortex, the ~57% of the VGF C-terminus immunoreactivity (Figure 4A) was found within the majority of parvoalbumin perikarya (Figure 4B), while the remained positive labeling stayed uncharacterized. The majority of the TLQP immunoreactivity (Figure 4C) was found in axons containing tyrosine hydroxylase (TH; Figure 4D) while roughly half of the TH positive nerve axons contained the TLQP peptides. In the nucleus accumbens, GGGE- (not shown) and VGF C-terminus- (Figures 4E,F) immunoreactivities were revealed in ~30%–40% of neuron terminals containing glutamic acid decarboxylase (GAD; and vice-versa) inside the shell. The N-terminus immunoreactivity was almost wholly found in all structures containing somatostatin (axons, perikarya and nerve terminals; Figures 4G,H) and in a small percentage (~5%) of NO synthase (NOS) containing cells (not shown).

## Discussion

Ample studies demonstrate that fragments derived from the VGF precursor have important neuronal functions or have been found in the CSF from neurological patients. In contrast, there is limited data regarding the expression and localization of the VGF-derived peptides in the brain (van den Pol et al., 1994). We have provided here the first evidence that rat brain is rich in fragments containing different portions of the proVGF sequence, and certain peptides are widely distributed at high levels in those brain areas with a key role in neuronal disorders, i.e., the prefrontal cortex, nucleus accumbens, hippocampus, hypothalamus and amygdaloid nucleus. Our results show that PCP treatment, which causes behavioral changes that closely mimic schizophrenia, estimated by us as a disruption of PPI in PCP-treated rats, induces also changes of VGF peptide levels in the prefrontal cortex and nucleus accumbens. Pertinent to this study is that the prefrontal cortex controls the activity of the nucleus accumbens (Mogenson et al., 1980; Grace et al., 2007). When we investigated the specific phenotype of the neurons containing the VGF peptides whose levels were changed after PCP treatment, we identified parvoalbumin- and TH- neurons (containing the VGF C-terminus and TLQP-peptides, respectively) within the prefrontal cortex, and GAD-, (containing the VGF C-terminus- and the GGGE-peptides), somatostatin- and NOS-neurons (containing the VGF N-terminus) in the nucleus accumbens. All these neurotransmitters and/or neuropeptides have a role in PCP related mechanisms. Indeed, it is noteworthy that PCP treatment induces not only behavioral changes, such as those that can be detected by assessing PPI, but also neuropathological changes, including changes related to dopaminergic and serotoninergic neurotransmissions, and reduction of parvoalbumin expression in the hippocampus and frontal cortex (Cochran et al., 2003; Reynolds et al., 2004; Abdul-Monim et al., 2006). GABA interneurons (in particular those containing parvoalbumin) of the prefrontal cortex are halted in PCP-treated animals (Mouri et al., 2007), with the consequence of disinhibiting (i.e., exciting) pyramidal neurons (see Moghaddam and Javitt, 2012). Somatostatin perfusion into the nucleus accumbens increases dopamine release in the same area, possibly via the glutamatergic system, through AMPA and NMDA receptors, or by inhibiting the GABA release (Ikeda et al., 2012). Disturbances in the parvoalbumin and somatostatin activity have been commonly reported in the prefrontal cortex from subjects with schizophrenia (Hashimoto et al., 2003; Mellios et al., 2009; Morris et al., 2009; Fung et al., 2010; Curley et al., 2011; Volk et al., 2012). Interestingly, we found that VGF peptides containing the N-terminus portion are decreased after PCP treatment in the nucleus accumbens, where we identified by MS analysis, the same N-terminal fragment (VGF_24–60_) previously found increased in the CSF from schizophrenia patients (Huang et al., 2006). These results highly suggest an involvement of the VGF N-terminal peptides in the alterations of the activity of the neuronal systems at the basis of schizophrenia. Regarding the GGGE peptides, previously identified and found reduced in the CSF from Alzheimer’s patients (Carrette et al., 2003), we revealed their presence in all the brain areas studied with a selective alteration in the prefrontal cortex after PCP treatment, suggesting their involvement in schizophrenia as well as Alzheimer mechanisms. Unfortunately, in this study, we failed to detect, by MS analysis, peptides containing the TLQP and GGGE sequences probably due to the low intensity of their ions. TLQP peptides were first identified in the rat brain (Trani et al., 2002) but despite their role in neuronal circuits (Thakker-Varia et al., 2007, 2010; Lin et al., 2014), they have never been identified by MS analysis, suggesting particular adversities to reveal them with this technique. However, the high levels of these peptides revealed by ELISA in the hypothalamus and hippocampus are in line with their known bioactivity in these areas (Hahm et al., 1999, 2002; Jethwa and Ebling, 2008). The role of the VGF peptides in neurons containing TH, parvoalbumin, GAD and somatostatin remains to be investigated; however the major hypothesized role is that VGF peptides could act as neuromodulators. The VGF role as neuromodulator has already been reported for certain VGF peptides, for instance NERPs regulate vasopressin secretion (Toshinai and Nakazato, 2009; D’Amato et al., 2012), TLQP-21 increases FSH release from the pituitary (Aguilar et al., 2013), TLQP-62 regulates hippocampal synaptic activity (Thakker-Varia et al., 2007, 2010; Bozdagi et al., 2008; Lin et al., 2015).

Regarding the significance of the specific VGF peptide changes we observed in the prefrontal cortex and nucleus accumbens, we attempt to make some speculations. In the prefrontal cortex, we found an increase in both VGF C-terminus and TLQP peptides after PCP treatment. Since at least in the hippocampus, the TLQP-62 enhances synaptic plasticity (Alder et al., 2003), we suggest that the parallel increase in the VGF C-terminus peptide/s (probably including the VGF precursor), as well as TLQP peptides, might be relevant for potentiating and/or increasing synaptic transmission. Our hypothesis would fit well with those studies showing a general increase in dendritic spine density after chronic PCP treatment in the rat prefrontal cortex (Flores et al., 2007). Hence, our VGF peptides could be one of the proteins involved in this process, as for instance the synapsin-1, which, involved in the neurotransmitter release at synapses, has been proved similarly up-regulated in the prefrontal cortex after PCP treatment (Pickering et al., 2013). As to the nucleus accumbens, GGGE and VGF C-/N- terminus levels were decreased after PCP treatment. All these peptides were reduced as a consequence of neurodegeneration in Alzheimer’s disease (Carrette et al., 2003; Cocco et al., 2010). In view of the significant reduction in the gray matter density observed in the nucleus accumbens after sub-chronic PCP treatment (Barnes et al., 2015), such findings may indicate a degree of neuronal degeneration in such area. In conclusion, increasing attention has been given recently to several neurotransmitters, in view of their role in schizophrenia related mechanisms. Since the prefrontal cortex and nucleus accumbens contain different neurotransmitters, it is important to investigate how they interact as a novel approach for the treatment of schizophrenia. The changes in the content of specific VGF peptides after PCP treatment found in this study, may suggest a role for these peptides acting as neuromodulators on the release of neurotransmitters crucial for neuropathology of schizophrenia, which may have a potential importance for therapeutic approaches. Future studies are needed to uncertain the VGF peptide roles in schizophrenia.

## Author Contributions

All authors had full access to all the data in the study and take responsibility for the integrity of the data and the accuracy of the data analysis. G-LF, CC, MRM, AA, FS and BN: study concept and design. BN, FS, CB, FD, BM, FV: acquisition of data. FS, BN, CC, MRM, CB and IM: analysis and interpretation of data. BN, CC, CB, FS and MRM: drafting of the manuscript. CC and MRM: critical revision of the manuscript for important intellectual content.

## Conflict of Interest Statement

The authors declare that the research was conducted in the absence of any commercial or financial relationships that could be construed as a potential conflict of interest.

## Footnotes

^1^http://us.expasy.org/tools
